# Supplementary material for: The Ki-67 and RepoMan mitotic phosphatases assemble via an identical, yet novel mechanism
Source: eLife. 2016 Aug 30;5:e16539. doi: 10.7554/eLife.16539 (PMC5005033; doi:10.7554/eLife.16539)
Supplement: Figure 1—source data 1. — DOI: http://dx.doi.org/10.7554/eLife.16539.003 [file elife-16539-fig1-data1.docx]

**Supplementary** **Table 1.** **Data collection and refinement statistics**

|  | RepoMan_383-423_:PP1γ_7-308_ | RepoMan_383-441_:PP1α_7-300_ | Ki-67_496-536_:PP1γ_7-308_ |
| --- | --- | --- | --- |
| **Data collection** |  |  |  |
| Space group | P6_1_ 2 2 | P2_1_ 2_1_ 2_1_ | P6_1_ |
| Cell dimensions |  |  |  |
| *a*, *b*, *c* (Å) | 86.4, 86.4, 215.9 | 160.8, 57.1, 73.6 | 90.8, 90.8, 206.7 |
| α, β, γ (°) | 90, 90, 120 | 90, 90, 90 | 90, 90, 120 |
| Resolution (Å) | 50.00 – 1.30 (1.32-1.30)* | 50.00 – 2.60 (2.64 – 2.60) | 39.33 – 2.00 (2.05 – 2.00) |
| *R*_merge_ | 4.9 (92.7) | 17.0 (60.5) | 9.2 (86.1) |
| *I* / σ*I* | 23.5 (2.4) | 15.3 (2.4) | 20.5 (3.1) |
| Completeness (%) | 100.0 (99.4) | 99.3 (90.4) | 99.9 (92.2) |
| Redundancy | 8.9 (8.6) | 11.5 (5.9) | 10.4 (10.3) |
|  |  |  |  |
| **Refinement** |  |  |  |
| Resolution (Å) | 37.4 – 1.3 | 46.5 – 2.6 | 39.3 – 2.0 |
| No. reflections | 221393 | 22041 | 64920 |
| *R*_work_ / *R*_free_ | 13.2/15.3 | 17.6/21.5 | 15.9/19.7 |
| No. atoms |  |  |  |
| Protein | 2780 | 5071 | 5161 |
| Ligand/ion | 44 | none | 15 |
| Water | 304 | 152 | 232 |
| *B*-factors |  |  |  |
| Protein | 17.1 | 29.7 | 28.0 |
| Ligand/ion | 27.9 | none | 27.5 |
| Water | 32.4 | 29.8 | 34.2 |
| R.m.s. deviations |  |  |  |
| Bond lengths (Å) | 0.008 | 0.002 | 0.007 |
| Bond angles (°) | 1.24 | 0.610 | 0.861 |

*Values in parentheses are for highest-resolution shell.
